# Thyroid and reproductive hormonal factors associated with menorrhagia among women in Kenya

**DOI:** 10.4102/ajlm.v14i1.2653

**Published:** 2025-04-10

**Authors:** Phidelis M. Marabi, Paul M. Kosiyo, Stanslaus K. Musyoki, Collins Ouma

**Affiliations:** 1 Department of Medical Laboratory Sciences, School of Health Sciences, Kisii University, Kisii, Kenya; 2 Department of Biomedical Sciences, Faculty of Public Health and Community Development, Maseno University, Maseno, Kenya; 3 Department of Medical Laboratory Sciences, Faculty of Medicine, Maseno University, Maseno, Kenya; 4 Department of Medical Laboratory Sciences, School of Health Sciences, South Eastern Kenya University, Kitui, Kenya

**Keywords:** menorrhagia, thyroid, reproductive, hormone, women, Kenya

## Abstract

**Background:**

Menorrhagia, characterised by menstrual blood loss exceeding 80 mL per cycle, is a common issue in Western Kenya. However, there are insufficient data on how hormonal disorders contribute to its occurrence.

**Objective:**

This study aimed to examine the differences and associations between thyroid and reproductive hormone levels in women with menorrhagia versus those without, in Bungoma County, Kenya.

**Methods:**

A comparative cross-sectional study was conducted among 428 women (214 with menorrhagia and 214 controls) aged 18–45 years, between 01 December 2022 and 31 September 2023 at Bungoma County Referral Hospital. The analysis included thyroid stimulating hormone, total and free triiodothyronine, thyroxine, follicle stimulating hormone (FSH), luteinising hormone, prolactin, oestrogen, progesterone, and testosterone.

**Results:**

Women experiencing menorrhagia had statistically significant increases in levels of FSH (*p* < 0.0001), oestrogen (*p* < 0.001), and total testosterone (*p* < 0.001), while prolactin levels had a statistically significant decrease (*p* < 0.001) compared to those without menorrhagia. There were no statistically significant differences in total triiodothyronine (*p* = 0.384), free triiodothyronine (*p* = 0.610), total thyroxine (*p* = 0.127), free thyroxine (*p* = 0.360), or thyroid stimulating (*p* = 0.118). No associations were found between menorrhagia and either thyroid or reproductive hormones.

**Conclusion:**

Elevated levels of FSH, oestrogen, and testosterone, along with reduced prolactin, may serve as potential biomarkers for diagnosing menorrhagia in premenopausal or reproductively aged women. A screening tool that integrates these hormonal markers could improve the accuracy of diagnosis and optimise treatment strategies in primary healthcare settings.

**What this study adds:**

The study suggests that levels of FSH, oestrogen, total testosterone, and prolactin differ significantly between women with and without menorrhagia, indicating their potential use in predicting the condition.

## Introduction

Menorrhagia, characterised by excessive or extended menstrual bleeding, may result from a variety of underlying conditions.^1^ It is estimated to affect approximately 11% – 13% of women during their reproductive years, with prevalence increasing with age.^2^ In several surveys, heavy menstrual bleeding remains one of the primary reasons women seek primary care.^3^ It is the fourth-most prevalent complaint that necessitates referral to a gynaecologist for evaluation and treatment.^4^ Menorrhagia has a profound impact on women’s physical, emotional, and social well-being, significantly reducing workplace productivity and overall quality of life.^5^ Additionally, it contributes to increased healthcare costs because of the need for frequent and sometimes expensive medical interventions.^2^

Hormonal imbalances are responsible for 80% of cases of abnormal uterine bleeding.^6^ Deficiency in thyroid hormones and irregularities in thyroid stimulating hormone (TSH) can impact ovarian function via multiple mechanisms, potentially altering follicle stimulating hormone (FSH) and luteinising hormone (LH), and affecting the metabolism of peripheral steroid hormones, prolactin, and sex hormone-binding globulin.^7^ Despite the known interaction between thyroid function and menstrual disorders, data specifically linking thyroid hormones with menorrhagia remain limited. Oestrogen and progesterone, the hormones that regulate the growth and shedding of the uterine lining during menstruation, must remain balanced to prevent excessive bleeding. Conditions such as polycystic ovarian syndrome, obesity, insulin resistance, or thyroid dysfunction can disrupt this balance, leading to heavy menstrual bleeding.^8^

Globally, the prevalence of thyroid disorders varies,^9^ with women being more frequently affected than men.^10^ Thyroid dysfunction, such as hyperthyroidism and hypothyroidism, can adversely affect women’s reproductive health.^11^ Hypothyroidism affects between 0.2% and 5.3% of the European population and between 0.3% and 3.7% of the United States population, depending on demographic factors.^12^ The prevalence of hyperthyroidism is reported to be 0.7% in Europe, 0.5% in the United States, and 0.3% in Australia.^9^ In South Africa, the incidence of hyperthyroidism among men is 0.00007 per 100 000, compared to a significantly higher rate of 0.009 among women.^13^ However, there are limited data on the prevalence of thyroid dysfunction in Kenya. Despite the global burden and recognised association between menstrual disorders and thyroid function, there is a lack of specific data on the link between thyroid hormones and menorrhagia in Kenya. While global statistics suggest that hormonal imbalances affect 80% of women,^14^ Kenyan local data are scarce.

In Kenya, while menorrhagia is estimated to affect around 48.9% of women,^15^ there remains a paucity of data on hormonal disorders among affected women. Studies in Western Kenya report a significant burden of menorrhagia, affecting 35.3% of women.^16^ Additionally, the Bungoma County Referral Hospital has reported a substantial number of gynaecological patients seeking care for menorrhagia.^17^ However, data on hormonal abnormalities in menorrhagic women remain scarce. Thus, this study explored the differences and associations in thyroid and reproductive hormone levels among women with and without menorrhagia at Bungoma County Referral Hospital, Kenya.

## Methods

### Ethical considerations

The study was approved by the Maseno University Scientific and Ethical Review Committee under reference number MUSERC/01166/22. Research authorisation was also granted by the National Commission for Science, Technology, and Innovation with approval number NACOSTI/P/22/22573. Additionally, data collection permission was obtained from Bungoma County Referral Hospital (reference number BDH/TP/B/VOL2). Written informed consent was obtained from all participants following a concise explanation of the study’s purpose. Participation in the study was entirely voluntary. Data security protocols included storing data in a secured physical location and using password protection for the computer. Participants’ personal information was kept confidential, with protocol numbers assigned for reference, and this information was used solely for research purposes.

### Study area

The study was conducted at Bungoma County Referral Hospital, located in Bungoma County, Western Kenya, between 01 December 2022 and 31 September 2023. Bungoma County Referral Hospital, a level 5 facility, serves as the primary referral health facility for the county and its surrounding areas, catering to a population of approximately 1 670 570 over a total area of 2206.9 km^2^.^18^ The gynaecological clinic at Bungoma County Referral Hospital serves approximately 6000 patients each year.^17^

### Study design and population

This research utilised a comparative cross-sectional design. The sample size was calculated using a formula for quantitative comparative studies, yielding a total of 428 participants (214 from each group). A systematic random sampling technique was employed, where every second individual who met the inclusion criteria was selected until the target sample size was reached.

### Inclusion criteria

The study aimed to recruit adult women aged 18 to 45 years who visited the clinic seeking treatment for menorrhagia at Bungoma County Referral Hospital. A comparison group was created by randomly selecting healthy women. A qualified gynaecologist assessed each patient’s menorrhagia, making a clinical diagnosis based on the definition of menorrhagia, which includes regular menstrual cycles with excessive blood loss (more than 80 mL per cycle or requiring changes of hygiene products more frequently than every 2 h) and/or a duration longer than 7 days.^19^

### Exclusion criteria

Participants were excluded if they had taken sylate, tranexamic acid, or any other medications for bleeding disorders in the last 3 months. Women receiving hormone replacement therapy, oral contraceptives, or other hormonal treatments that could affect menstrual bleeding or hormonal levels, were also disqualified. Those with diagnosed thyroid disorders (such as hyperthyroidism or hypothyroidism) or chronic illnesses (including diabetes, hypertension, or significant cardiovascular diseases) were not eligible. Additionally, women with pelvic conditions (such as uterine fibroids, polyps, or cancers) that might influence bleeding patterns, as well as those who had recent gynaecological surgery affecting menstrual bleeding, were excluded. Participants with blood disorders, such as haemophilia or thrombocytopenia, were also disqualified from the study.

### Sample and data collection

Participants were divided into menorrhagic and non-menorrhagic groups based on a screening method as previously employed,^20^ which included self-reported menstrual blood loss and clinical evaluation. This ensured a standardised and reproducible approach to participant selection. A structured questionnaire was used to collect sociodemographic and clinical information, such as age, residence, parity, occupation, and literacy level. Blood samples were collected in a plain vacuum tube (Becton, Dickinson and Company, Franklin Lakes, New Jersey, United States). The samples were promptly inverted gently and assigned a unique identification number for each participant. They were then quickly transported to the laboratory. In the laboratory, the samples were centrifuged at 3000 revolutions per minute for 5 min to isolate the serum within 4 h of collection. If immediate testing was not feasible, the separated samples were stored at 4 °C for a maximum of 24 h.

### Laboratory analyses

The thyroid and reproductive hormone profiles were evaluated using the MAGLUMI 800 CLIA instrument (Shenzhen New Industries Biomedical Engineering Co. [Snibe] Diagnostics, Shenzhen, China). The analysis included TSH, total and free triiodothyronine, thyroxine, FSH, LH, prolactin, oestrogen, progesterone, and testosterone. The analyser uses the luminescence principle for clinical chemistry.

The reference ranges for thyroid and reproductive hormones in adult women were obtained from the laboratory at Bungoma County Referral Hospital: total triiodothyronine levels: 60 ng/dL – 180 ng/dL; free triiodothyronine levels: 130 pg/dL – 450 pg/dL; total thyroxine levels: 5.0 µg/dL – 12.0 µg/dL; free thyroxine levels: 0.7 ng/dL – 1.9 ng/dL; TSH levels: 0.5 milli-International Units (mIU)/L – 2.87 mIU/L; FSH levels: 1.5 IU/L – 12.4 IU/L; LH levels: 5 IU/L – 25 IU/L; prolactin levels: 2 ng/mL – 29 ng/mL; oestrogen levels: 30 pg/mL – 400 pg/mL; progesterone levels: 5 ng/mL – 20 ng/mL; and testosterone levels: 0.5 nmol/L – 2.4 nmol/L. Reagents, control materials and standards were stored at 2 °C – 4 °C and allowed to reach room temperature prior to testing, ensuring thorough mixing according to the manufacturer’s guidelines. Control materials with both normal and pathological levels were analysed before the sample testing commenced.

### Data analysis

The analysis was conducted using the Statistical Package for Social Science (v.26) software (IBM Corporation, Armonk, New York, US). Descriptive statistics, such as frequency, median, and dispersion, were presented in tables. The Shapiro-Wilk test was employed to assess normality, while histograms and box plots were used for visual confirmation of the data distribution. The Chi-square test was applied for categorical descriptive data, including residence, parity, and occupation. The Mann-Whitney *U* test was used to compare continuous descriptive data, such as age. Since the data did not adhere to a normal distribution, the Mann-Whitney *U* test was also utilised to compare thyroid and reproductive hormone levels between menorrhagic and non-menorrhagic women. To assess the relationship between menorrhagia and hormonal parameters, binary logistic regression was conducted, as the dependent variable was categorical (menorrhagic vs non-menorrhagic). Participants with potential confounding factors, such as body mass index, dietary factors, and other variables that could influence hormonal levels or menstrual patterns, were excluded from the analysis. A *p*-value of ≤ 0.05 was considered statistically significant.

## Results

### Demographic characteristics of the study population

A total of 428 adult women aged 18 years to 45 years (median [interquartile range]: 33.5 [11.0] years) were included in the study (Table 1). Among them, there were 214 participants with menorrhagia and an equal number of healthy non-menorrhagic women. No significant differences were observed between the groups regarding urban or rural residence (*p* = 0.139), parity (*p* = 0.390), and occupation (*p* = 0.846) (Table 1).

**TABLE 1:** Demographic characteristics of the study participants at Bungoma County Referral Hospital, Kenya, December 2022 – September 2023.

Demographic characteristic	Menorrhagic women (cases; *n* = 214)				Non-menorrhagic women (controls; *n* = 214)				*p*
	*n*	%	Median	IQR	*n*	%	Median	IQR	
Age	-	-	34.0	6.0	-	-	33.0	14.0	0.209†
**Residence**	-	-	-	-	-	-	-	-	0.139‡
Rural	136	63.6	-	-	120	56.0	-	-	-
Urban	78	36.4	-	-	94	44.0	-	-	-
**Parity**	-	-	-	-	-	-	-	-	0.390‡
Parous	177	82.7	-	-	169	79.0	-	-	-
Non-parous	37	17.3	-	-	45	21.0	-	-	-
**Occupation**	-	-	-	-	-	-	-	-	0.846‡
Formally employed	102	47.7	-	-	99	46.3	-	-	-
Informally employed	112	52.3	-	-	115	53.7	-	-	-
**Literacy**	-	-	-	-	-	-	-	-	N/A
Literate	214	100.0	-	-	214	100.0	-	-	-
Not literate	Nil	Nil	-	-	Nil	Nil	-	-	-

Note: The data are presented as frequency and percentage unless stated otherwise. The frequency and proportion of five demographic characteristics were summarised for both menorrhagic and non-menorrhagic participants. Statistical significance was assessed using the Mann-Whitney *U* test for continuous variables, while the chi-square test was used to determine the significance of differences for categorical variables.

Nil, no demographic characteristic was observed in either the menorrhagic or non-menorrhagic groups; N/A, the *p*-value was not calculated; IQR, interquartile range.

† , statistical significance was determined using Mann–Whitney *U* test;

‡ , statistical significance of the differences was determined using the chi-square test.

### Comparison of thyroid hormones between menorrhagic and non-menorrhagic women

There were no significant differences between menorrhagic and non-menorrhagic women regarding total triiodothyronine (*p* = 0.384), free triiodothyronine (*p* = 0.610), total thyroxine (*p* = 0.127), free thyroxine (*p* = 0.360), and TSH levels (*p* = 0.118) (Table 2).

**TABLE 2:** Comparison of thyroid hormones between menorrhagic and non-menorrhagic women, Bungoma County Referral Hospital, Kenya, December 2022 – September 2023.

Parameter	Menorrhagic women (cases; *n* = 214)				Non-menorrhagic women (controls; *n* = 214)				*p*
	*n*	%	Median	IQR	*n*	%	Median	IQR	
**Total triiodothyronine (ng/dL)**	-	-	124.00	60.25	-	-	119.00	53.20	0.384
Normal reference range: 60–180	210	98.1	-	-	213	95.3	-	-	-
> 180	4	1.9	-	-	1	0.5	-	-	-
< 60	0	0.0	-	-	0	0.0	-	-	-
**Free triiodothyronine (pg/dL)**	-	-	264.50	182.25	-	-	266.00	201.50	0.610
Normal reference range: 130–450	193	90.2	-	-	209	97.7	-	-	-
> 450	12	5.6	-	-	0	0.0	-	-	-
< 130	9	4.2	-	-	5	2.3	-	-	-
**Total thyroxine (g/dL)**	-	-	9.24	3.82	-	-	8.95	4.17	0.127
Normal reference range: 5.0–12.0	206	96.3	-	-	211	98.6	-	-	-
> 12.0	8	3.7	-	-	1	0.5	-	-	-
< 5.0	0	0.0	-	-	2	0.9	-	-	-
**Free thyroxine (ng/dL)**	-	-	1.32	0.55	-	-	0.51	0.51	0.360
Normal reference range: 0.7–1.9	201	93.9	-	-	198	92.5	-	-	-
> 1.9	13	6.1	-	-	4	1.9	-	-	-
< 0.7	0	0.0	-	-	12	5.6	-	-	-
**TSH (mIU/L)**	-	-	1.37	1.01	-	-	1.61	1.40	0.118
Normal reference range: 0.45–2.87	209	97.7	-	-	204	95.3	-	-	-
> 2.87	0	0.0	-	-	0	0.0	-	-	-
< 0.45	5	2.3	-	-	10	4.7	-	-	-

Note: The data are expressed as median values with interquartile ranges for thyroid hormones unless indicated otherwise. Statistical significance was assessed using the Mann-Whitney *U* test, with a *p*-value of ≤ 0.05 considered statistically significant. There were no significant differences in total triiodothyronine, free triiodothyronine, total thyroxine, free thyroxine, and TSH levels between menorrhagic and non-menorrhagic women.

IQR, interquartile range; TSH, thyroid stimulating hormone.

### Comparison of reproductive hormones between menorrhagic and non-menorrhagic women

Menorrhagic women were more likely to have increased FSH (59/214; 27.6%), oestrogen (214/214; 100%) and testosterone (214/214; 100%) levels than non-menorrhagic women (FSH: 10/214, 4.7%; oestrogen: 186/214, 86.5%; testosterone: 210/214, 98.1%) (Table 3).

**TABLE 3:** Comparison of reproductive hormones between menorrhagic and non-menorrhagic women, Bungoma County Referral Hospital, Kenya, December 2022 – September 2023.

Parameter	Menorrhagic women (cases; *n* = 214)				Non-menorrhagic women (controls; *n* = 214)				*p*
	*n*	%	Median	IQR	*n*	%	Median	IQR	
**FSH (IU/L)**	-	-	9.39	7.30			3.84	2.41	< 0.0001*
Normal reference range: 1.5–12.4	153	71.5	-	-	192	89.7	-	-	-
< 1.5	2	0.9	-	-	12	5.6	-	-	-
> 12.4	59	27.6	-	-	10	4.7	-	-	-
**LH (IU/L)**	-	-	8.06	5.48	-	-	7.64	2.88	0.4270
Normal reference range: 5–25	178	83.2	-	-	214	100.0	-	-	-
< 5	0	0.0	-	-	0	0.0	-	-	-
> 25	36	16.8	-	-	0	0.0	-	-	-
**Prolactin (ng/dL)**	-	-	7.51	6.51	-	-	12.35	11.97	< 0.0001*
Normal reference range: 2–25	198	92.5	-	-	207	96.7	-	-	-
< 2	16	7.5	-	-	7	3.3	-	-	-
> 25	0	0.0	-	-	0	0.0	-	-	-
**Oestrogen (pg/dL)**	-	-	86.80	68.75	-	-	59.60	38.20	< 0.0001*
Normal reference range: 30–400	214	100.0	-	-	186	86.9	-	-	-
< 30	0	0.0	-	-	28	13.1	-	-	-
> 400	0	0.0	-	-	0	0.0	-	-	-
**Progesterone (ng/mL)**	-	-	7.97	5.96	-	-	7.43	2.97	0.0940
Normal reference range: 5–20	180	84.1	-	-	207	96.7	-	-	-
< 5	32	15.0	-	-	7	3.3	-	-	-
> 20	2	0.9	-	-	0	0.0	-	-	-
**Testosterone (nmol/L)**	-	-	0.89	0.30	-	-	0.61	0.07	< 0.0001*
Normal reference range: 0.5–2.4	214	100.0	-	-	210	98.1	-	-	-
< 0.5	0	0.0	-	-	4	1.9	-	-	-
> 2.4	0	0.0	-	-	0	0.0	-	-	-

Note: The data are presented as median values with interquartile ranges for reproductive hormones unless stated otherwise. The Mann-Whitney *U* test was used to assess statistical significance, with a *p*-value of ≤ 0.05 considered statistically significant.

IQR, interquartile range; LH, luteinising hormone; FSH, follicle stimulating hormone.

* , statistically significant.

Menorrhagic women (16/214; 7.5%) were more likely than non-menorrhagic women (7/214; 3.3%); to have reduced prolactin levels (Table 3).

### Association between thyroid hormones and menorrhagia

Menorrhagia was not associated with total triiodothyronine (odds ratio [OR] = 1.001, 95% confidence interval [CI] = 0.996 – 1.006, *p* = 0.541), free triiodothyronine (OR = 1.001, 95% CI =0.999 – 1.002, *p* = 0.371), total thyroxine (OR = 1.080, 95% CI = 0.993 – 1.175, *p* = 0.092), free thyroxine (OR = 1.467, 95% CI = 0.845–2.561, *p* = 0.233), and TSH (OR = 0.891, 95% CI = 0.705 – 1.25, *p* = 0.390) (Table 4).

**TABLE 4:** Association between menorrhagia and thyroid hormones, Bungoma County Referral Hospital, Kenya, December 2022 – September 2023.

Parameter	Women category	OR	95% CI	*p*
**Total triiodothyronine (ng/dL)**				
60–180	Non-menorrhagia	Ref.	-	-
> 180	Menorrhagia	1.001	0.996–1.006	0.541
**Free triiodothyronine (pg/dL)**				
130–450	Non-menorrhagia	Ref.	-	-
> 450	Menorrhagia	1.001	0.999–1.002	0.371
**Total thyroxine (g/dL)**				
5.0–12.0	Non-menorrhagia	Ref.	-	-
> 12.0	Menorrhagia	1.080	0.993–1.175	0.092
**Free thyroxine (ng/dL)**				
0.7–1.9	Non-menorrhagia	Ref.	-	-
> 1.9	Menorrhagia	1.467	0.845–2.561	0.233
**TSH (mIU/L)**				
0.5–2.87	Non-menorrhagia	Ref.	-	-
> 2.87	Menorrhagia	0.891	0.705–1.125	0.390

Note: Women (*n* = 428) were categorised according to the presence or absence of menorrhagia. Binary logistic regression analysis was conducted to obtain the odds ratio (OR) and 95% confidence intervals (CI). A *p*-value of ≤ 0.05 was considered statistically significant. In this analysis, women without menorrhagia were used as the reference group.

CI, confidence interval; OR, odds ratio; TSH, thyroid stimulating hormone.

### Association between reproductive hormones and menorrhagia

Menorrhagia was not associated with FSH (OR = 0.849, 95% CI = 0.712 – 1.013, *p* = 0.069), LH (OR = 1.021, 95% CI = 0.808 – 1.291, *p* = 0.860), prolactin (OR = 1.202, 95% CI = 0.984 – 1.469, *p* = 0.071), oestrogen (OR = 1.015, 95% CI = 0.988– 1.043, *p* = 0.279), or progesterone (OR = 0.951, 95% CI = 0.749 – 1.207, *p* = 0.678). However, the model did not determine the association between total testosterone and menorrhagia (Table 5).

**TABLE 5:** Association between menorrhagia and reproductive hormones, Bungoma County Referral Hospital, Kenya, December 2022 – September 2023.

Parameter	Women category	OR	95%CI	*p*
**FSH (IU/L)**				
1.5–12.4	Non-menorrhagia	Ref.	-	-
> 12.4	Menorrhagia	0.849	0.712–1.013	0.069
**LH (IU/L)**				
5–25	Non-menorrhagia	Ref.	-	-
25.0	Menorrhagia	1.021	0.808–1.291	0.860
**Prolactin (ng/dL)**				
2–29	Non-menorrhagia	Ref.	-	-
< 2	Menorrhagia	1.202	0.984–1.469	0.071
**Oestrogen (pg/dL)**				
30–400	Non-menorrhagia	Ref.	-	-
< 30	Menorrhagia	1.015	0.988–1.043	0.279
**Progesterone (ng/mL)**				
5–20	Non-menorrhagia	Ref.	-	-
> 20	Menorrhagia	0.951	0.749–1.207	0.678
**Testosterone (nmol/L)**				
0.5–2.4	Non-menorrhagia	Ref.	-	-
> 2.4	Menorrhagia	N/A	N/A	N/A

Note: A total of 428 women were categorised according to whether they experienced menorrhagia. To determine the odds ratio (OR) and 95% confidence intervals (CI), binary logistic regression analysis was performed. A *p*-value of ≤ 0.05 was considered statistically significant. In this analysis, the reference group consisted of women who did not have menorrhagia.

CI, confidence interval; OR, odds ratio; LH, luteinising hormone; N/A, indicates that the model could not establish an association; FSH, follicle stimulating hormone.

## Discussion

In this study, we found that menorrhagic women exhibited statistically significant, increased levels of FSH, oestrogen, and testosterone, while prolactin levels were statistically significantly lower compared to non-menorrhagic women. However, no statistically significant differences were observed in thyroid hormone levels, and none of the evaluated hormonal parameters had statistically significant associations with menorrhagia.

In the current study, menorrhagic women had an identical median age to non-menorrhagic women. The median age of all participants in the study was 33.5 years, which was closely similar to the study population of a 2018 study done in Pakistan, whose mean age was 35.56 years.^21^ However, the age range in the current study differed from one conducted in Tanzania, published in 2022,^22^ which focused on the age range of 15 to 59 years.

In this study, the living environments (rural or urban) of women with menorrhagia did not significantly differ from those of women without menorrhagia, a finding that aligns closely with research conducted in Tanzania and published in 2022.^22^ The present study revealed that a greater percentage of women with menorrhagia reported having had at least one child, although no significant difference in parity was observed. These results contrast with those from a study conducted in the United States, published in 2016,^23^ where only one person was parous. The difference may be because of the sample size, with the latter study involving only 38 participants. The present study revealed that a significant proportion of women with menorrhagia were engaged in informal employment, showing no notable difference compared to women without menorrhagia. These results align with those of a study conducted in Tanzania and published in 2022,^22^ which reported that 52.5% of women with menorrhagia are employed as farmers.

This study indicated that levels of total triiodothyronine, free triiodothyronine, total thyroxine, free thyroxine, and TSH were similar between menorrhagic and non-menorrhagic women. Additionally, it found no significant association between total triiodothyronine, free triiodothyronine, total thyroxine, and free thyroxine hormones and menorrhagia. The current findings contrast with those of an Egyptian study, published in 2006,^24^ which identified a notable difference in the levels of total triiodothyroninee, free triiodothyronine, total thyroxine, and TSH between women with menorrhagia and the control group. The differences in the results may be a result of variations in the study populations concerning geography and ethnicity. The findings also contradict those of an Iraqi study, published in 2019,^25^ which found that as thyroxine reduces and TSH increases, the likelihood of menorrhagia is increased. However, the current study found that 1.9% of menorrhagic women had total triiodothyronine levels that were greater than the expected reference range. About 5.6% of menorrhagic women had higher levels of free triiodothyronine, whereas 4.2% had lower levels below the anticipated reference range. Some 3.7% of menorrhagic women had total thyroxine levels that exceeded the expected reference range. About 6.1% of menorrhagic women had higher levels of free thyroxine than the anticipated reference range, whereas 2.3% had lower TSH. The findings were consistent with those of an Indian study, published in 2015,^26^ which showed that 9% of patients had hypothyroidism and 3% had hyperthyroidism. The present findings can be explained by the fact that the onset of hypothyroidism or hyperthyroidism is so gradual that characteristic clinical manifestations may not present for months or years. Furthermore, in hypothyroid women, menorrhagia may be the only presenting complaint.^27^ The abnormal thyroid hormone levels (both total and free triiodothyronine and thyroxine) observed in a small percentage of menorrhagic women in this study suggest that thyroid dysfunction may be an underlying factor contributing to this condition. Clinicians should consider routine thyroid function tests for women presenting with menorrhagia to identify potential endocrine contributors. Understanding the thyroid status of menorrhagic patients may guide treatment decisions.^28^ For instance, if hypothyroidism or hyperthyroidism is diagnosed, appropriate management of thyroid disease could potentially alleviate menorrhagic symptoms and improve overall menstrual health.^29^ The findings align with previous studies, indicating a possible commonality in the prevalence of thyroid dysfunction among women with menorrhagia.^28^ This consistency suggests that similar screening practices may be beneficial across different populations and healthcare settings. The present findings highlight the need for further research to explore the causal relationship between thyroid disorders and menorrhagia. Understanding whether thyroid dysfunction directly contributes to menstrual irregularities could enhance clinical approaches and treatment strategies. The present study was, however, limited in the sense that being a cross-sectional study, the present study did not assess how thyroid hormonal parameters change over time in relation to menorrhagia. Future longitudinal studies on how thyroid hormones change over time in relation to menorrhagia are recommended so as to help understand how these hormones evolve over time and their impact on treatment outcomes. In light of the present findings, public health initiatives that focus on increasing awareness of thyroid health in relation to menstrual disorders, potentially leading to earlier diagnosis and intervention, are recommended. The present findings further suggest that women experiencing menorrhagia may benefit from a comprehensive evaluation that includes hormonal profiling to tailor treatment options based on their specific endocrine status.

In the current study, FSH levels were considerably greater in menorrhagic patients compared to non-menorrhagic cases, with around 27.6% of menorrhagic cases having raised FSH levels and 0.9% having lower FSH levels than the normal range. It was additionally found that FSH levels were not significantly associated with menorrhagia. This lack of association may be attributed to the multifactorial nature of menorrhagia, which is influenced by a combination of hormonal, haematological, and structural factors. While FSH plays a crucial role in ovarian function, excessive menstrual bleeding is more commonly driven by imbalances in oestrogen and progesterone, abnormalities in endometrial haemostasis, or underlying uterine conditions such as fibroids and adenomyosis.^30^ The interplay of these factors may overshadow the role of FSH in determining bleeding severity. The current results are consistent with those of a study conducted in Egypt, published in 2006,^24^ that found a substantial difference in FSH levels between menorrhagic women and the control group. The current findings are also in agreement with those reported in a study by Eldred and Thomas, published in 1994,^31^ that demonstrated no significant differences in the plasma concentrations of FSH between those with objectively heavy loss and those with normal loss. These present findings may be explained by the fact that shorter menstrual cycles could be an early sign of a woman entering the menopausal transition, while longer cycles tend to appear later, often associated with elevated FSH levels, which suggest a decrease in active ovarian follicles.^32^ Clinically, elevated FSH levels in menorrhagic women in the present study could serve as a potential marker for identifying women at risk for heavy menstrual bleeding. However, since FSH is not significantly associated with the severity of menorrhagia, it may not be reliable for diagnosing the condition itself. From a clinical perspective, the elevated FSH levels in menorrhagic women might suggest a potential endocrine disruption, prompting clinicians to explore hormonal treatments or further investigate ovarian function in these patients.^33^ The corroboration of the current findings with studies from different populations (like the Egyptian study)^24^ reinforces the idea that menorrhagia might be associated with hormonal imbalances across diverse groups, which could have implications for global health approaches to managing menstrual disorders. The lack of significant association with the condition in the current study emphasises the need for a broader understanding of the aetiology of menorrhagia.

The present data revealed significantly lower prolactin levels in the menorrhagic group compared to the non-menorrhagic group, with about 7.5% of menorrhagic patients exhibiting reduced prolactin levels. It further demonstrated no significant association between prolactin and the likelihood of experiencing menorrhagia. This lack of association may be because of variations in individual physiological responses to hormonal fluctuations. Some women with altered prolactin levels may exhibit compensatory endocrine mechanisms that help maintain normal menstrual function, thereby masking a direct relationship with menorrhagia. Additionally, factors such as stress, medication use (e.g. dopamine agonists or antagonists), and body mass index can influence prolactin levels, contributing to variability in findings.^34^ However, these factors were not evaluated in the present study. These results are consistent with those of a study conducted in Egypt, published in 2006,^24^ which showed a significant difference in prolactin levels between menorrhagic and non-menorrhagic women. The present findings, however, contradict an Indian study, published in 2017,^35^ that demonstrated higher prolactin levels in cases. The reduced prolactin levels observed in this study may be due to the lower TSH levels in the menorrhagic group, as previous research has established a connection between TSH and prolactin levels.^35^ The lack of significant association between prolactin levels and the likelihood of experiencing menorrhagia implies that clinicians should not rely on prolactin measurements alone when assessing patients with heavy menstrual bleeding. This could shift the diagnostic focus towards other hormonal or systemic factors, such as thyroid function (given the noted link between TSH and prolactin levels). The association of lower TSH and prolactin levels in the menorrhagic group could suggest a broader endocrine imbalance, and the interrelationship between TSH and prolactin could be an avenue for further exploration. Clinicians might consider evaluating thyroid function more closely in patients presenting with menorrhagia. The discrepancies between the current findings and other studies (e.g. the Egyptian study^24^ showing higher prolactin levels, and the Indian study^35^) highlight the need for further research to understand the diverse physiological mechanisms behind menorrhagia in different populations. This variability may be influenced by genetic, environmental, or lifestyle factors. The present findings underscore the complexity of hormonal interactions in menorrhagia and suggests a need for a more nuanced approach to diagnosis and treatment that considers individual patient profiles rather than relying solely on prolactin levels. Further research is warranted to clarify these relationships and guide clinical practice.

In the present study, oestrogen levels demonstrated significantly higher levels in menorrhagic cases compared to non-menorrhagic cases. The findings contradicted the findings of Eldred and Thomas’s study, published in 1994,^31^ which demonstrated no significant difference between those with heavy blood loss and those with normal bleeding. The lack of association observed in our study may be due to individual variations in endometrial receptor sensitivity to oestrogen. Some women may exhibit heightened endometrial responsiveness to circulating oestrogen, leading to excessive proliferation and bleeding, while others may experience resistance, reducing oestrogen’s impact on menstrual flow.^36^ Additionally, other factors such as coagulation disorders, vascular abnormalities, and inflammatory mediators could contribute to menorrhagia independently of oestrogen levels, potentially masking a direct hormonal effect.^37^ The findings differ from those of a study conducted in Egypt, published in 2006,^24^ that showed reduced oestrogen in menorrhagic cases compared to the control group. This discrepancy highlights the need for further research to clarify the role of oestrogen in menorrhagia, as well as potential variations in population characteristics, methodologies, or underlying conditions that could influence these outcomes. This finding may be explained by the fact that unopposed oestrogen makes the endometrium fragile, highly vascular, and lacking adequate stromal support, leading to prolonged and excessive uterine bleeding.^19^ This insight can inform both preventive and therapeutic strategies, suggesting that managing oestrogen levels might mitigate bleeding in affected individuals. The higher oestrogen levels in menorrhagic cases in the present study suggest that hormonal imbalance, specifically elevated oestrogen, may play a crucial role in the pathophysiology of heavy menstrual bleeding. This could help identify hormonal factors that contribute to menorrhagia, enhancing diagnostic accuracy and treatment options. Understanding the hormonal dynamics involved in menorrhagia could guide clinicians in choosing appropriate treatments, such as hormonal therapies that balance oestrogen and progesterone, potentially reducing bleeding episodes and improving patient outcomes. These findings not only contribute to the understanding of the hormonal underpinnings of menorrhagia but also underscore the importance of further investigation into the mechanisms and potential treatments for this condition.

In this study, testosterone levels were notably elevated in menorrhagic women compared to those without menorrhagia. However, no significant association was found between testosterone and menorrhagia. This lack of association may be attributed to the complex, multifactorial nature of menorrhagia, where various hormonal and non-hormonal factors interact to influence menstrual bleeding. While elevated testosterone levels could indicate a hormonal imbalance contributing to menstrual disorders, other underlying factors such as ovarian dysfunction, endometrial pathology, or co-existing conditions may obscure the relationship between testosterone and menorrhagia.^6^ These findings are consistent with an Egyptian study, published in 2006,^24^ which also reported higher testosterone levels in the menorrhagic group compared to the non-menorrhagic group. The findings also concur with Redmond’s^38^ study, published in 2004, that showed elevated testosterone levels in the menorrhagic group relative to the non-menorrhagic group. In the current study, elevated testosterone levels in the menorrhagic group could be linked to slightly elevated LH levels in the same group, as prior research has shown that LH influences androgen synthesis by theca cells.^39^ In the 1970s, it was found that theca cells in developing follicles synthesised androgens (including androstenedione, testosterone, and dihydrotestosterone) in response to LH. Additionally, it was noted that androgens produced by theca cells were transformed into oestradiol by the aromatase (CYP19A1) enzyme within granulosa cells.^39^ The present findings highlight the need for additional studies to explore the mechanisms by which testosterone and LH interact in the context of menorrhagia. Future research could examine the role of theca cell function and aromatase activity, potentially identifying new biomarkers for diagnosis or targets for therapy. The higher testosterone levels observed in menorrhagic women in this study may suggest a hormonal imbalance that could be linked to menstrual disorders. Understanding this relationship could help clinicians identify potential hormonal causes of menorrhagia in affected patients. Although the study found no significant association between testosterone and menorrhagia, the elevated testosterone levels observed in menorrhagic patients might still serve as a potential biomarker for further investigation. Clinicians might consider evaluating testosterone levels in patients presenting with heavy menstrual bleeding, particularly if other symptoms of hormonal imbalance are present. The elevated testosterone levels in menorrhagic individuals, alongside slightly increased LH levels, may suggest a dysregulation in the hypothalamic-pituitary-gonadal axis. Understanding this relationship is crucial for clinicians as it points to potential underlying endocrine disorders that could contribute to menorrhagia. Recognising that elevated testosterone levels may be part of the pathophysiology of menorrhagia could open avenues for targeted therapies. For example, interventions that modulate androgen levels or LH action may provide new options for managing symptoms in affected individuals.

### Recommendations

This study recommends developing a risk assessment tool for menorrhagia in primary healthcare settings, integrating hormonal markers such as FSH (> 10 IU/L), oestradiol outside normal cycle ranges, total testosterone (> 70 ng/dL), and prolactin (> 25 ng/mL). These thresholds, alongside clinical symptoms, would help identify at-risk women for early intervention.

### Limitations

Being a cross-sectional study, the present study did not assess how hormonal parameters change over time in relation to menorrhagia.

### Conclusion

Follicle stimulating hormone, prolactin, oestradiol, and testosterone are potential markers for menorrhagia. Although no direct associations were found, the significant hormonal differences suggest that monitoring these markers could help identify hormonal imbalances and guide targeted treatment, improving early detection and management of menorrhagia.
